# Response of a Benthic *Sargassum* Population to Increased Temperatures: Decline in Non-Photochemical Quenching of Chlorophyll a Fluorescence (NPQ) Precedes That of Maximum Quantum Yield of PSII

**DOI:** 10.3390/plants14050759

**Published:** 2025-03-01

**Authors:** Ricardo M. Chaloub, Rodrigo Mariath V. da Costa, João Silva, Cristina A. G. Nassar, Fernanda Reinert, Maria Teresa M. Széchy

**Affiliations:** 1 Department of Biochemistry, Institute of Chemistry, Federal University of Rio de Janeiro, Rio de Janeiro 21941-909, Brazil; 2 Research Institute Botanical Garden of Rio de Janeiro, Rio de Janeiro 22460-030, Brazil; rodrigo.costa@usu.edu.br; 3 Santa Úrsula University, Rio de Janeiro 22231-040, Brazil; 4 Centre of Marine Sciences (CCMAR), University of Algarve, 8005-139 Faro, Portugal; jmsilva@ualg.pt; 5 Department of Botany, Institute of Biology, Federal University of Rio de Janeiro, Rio de Janeiro 21941-902, Brazil; cagnassar@hotmail.com (C.A.G.N.); fernandareinert@gmail.com (F.R.); mtmszechy@gmail.com (M.T.M.S.)

**Keywords:** brown algae, photosynthesis, PAM fluorometry, rapid light curves (RLCs), complementary quantum yields

## Abstract

*Sargassum* is an important primary producer of rocky bottom communities in coastal ecosystems. Like other parts of the planet, benthic populations of *S. natans* from Ilha Grande Bay (IGB), southeastern Brazil, have been suffering from different forms of natural and anthropogenic disturbances, in particular increasing seawater temperatures. The aim of this study was to understand the effects of temperature on the photosynthetic performance of *S. natans* using the pulse amplitude modulated (PAM) fluorometry. In the field experiments, the occurrence of photoprotection resulted in a difference between the effective and maximum quantum yields [(*ΔF (F’_m_ − F_s_)/F’_m_* and *F_v_/F_m_*, respectively) that was maximized at noon. The stress induced by incubation at 32–35 °C caused a decrease in *F_v_/F_m_* by 33% on the first day and approximately 20% on subsequent days. In the laboratory, using two co-occurred species of *S. natans* and *Padina gymnospora*, we verified that the photosynthetic apparatus of *S. natans* collapses at 34 °C. The fate of the energy absorbed by photosystem II (PSII) antenna showed that, in S. natans, photochemical activity and non-photochemical quenching of chlorophyll fluorescence (NPQ) drastically decrease, and only the passive dissipation in the form of heat and fluorescence remains. Our results indicate the disappearance of the NPQ photoprotection at 34 °C before the decline of *F_v_/F_m_* as the reason for the collapse of photochemistry of *Sargassum*.

## 1. Introduction

The brown macroalgal genus *Sargassum* (Sargassaceae, Fucales) is one of the most important habitat-forming taxa of shallow coastal environments, occurring along the tropical and warm temperate regions worldwide [1]. *Sargassum* beds represent several resources for benthic communities, such as food and space for reproduction, nursery, and protection for the associated fauna [2,3]. As a whole, fucoid forests are important because they occupy “large areas, bioregions, and ecosystems, provide ecological functions such as high productivity, biodiversity, and habitat for iconic and endemic species, and support a variety of ecosystem services, like commercial fisheries, regulation of nutrients and carbon, and cultural values” [4]. Considering the actual scenario regarding climate and oceanographic events, a deep understanding of the photosynthetic performance of *Sargassum* under the impact of increasing surface seawater temperatures (SSTs) is critical for forecasting their responses undergoing global warming, providing better conservation procedures. The observed current ecosystems disruptions, due to global climate changes or local anthropogenic disturbances, deeply affect the survivorship, growth, reproduction, and distribution of marine organisms [4], particularly for sessile species living on shallow rocky bottoms, such as macroalgae [5].

There are relevant laboratory and field studies on the effects of temperature and other abiotic factors on the physiology and growth of *Sargassum* species from different regions at short temporal scales [6,7,8,9,10]. These studies indicate a negative effect of increasing temperature on *Sargassum* species, particularly at temperatures above 30 °C for species in warm-temperate regions and 33 °C for tropical species [10,11]. The negative effects of increased temperatures on *Sargassum* populations are reported by long-term studies. For instance, the frequency of occurrence and the relative cover of *Sargassum* from populations subjected to heated effluent declined after more than two decades of operation of the Brazilian Nuclear Power Station (BNPS). Yet, the abundance of other brown algal species from that region, particularly *Padina gymnospora* (Dictyotales), remained high during the same period [12,13]. The decline and disappearance of *Sargassum* and other Fucales subjected to increasing temperatures are described for other regions of the world [14,15]. Elevated seawater temperature directly or indirectly alters the photosynthetic performance of algae [16], affecting their primary production.

Photosynthesis is highly sensitive to high temperatures and is often inhibited before other cellular functions are impaired due to the damage to the PSII and the inhibition of Rubisco activity [17,18,19,20]. The effects of increasing temperature, as well as the combined effects of temperature and irradiance on photosynthetic activity and the thermal tolerance of different *Sargassum* species, have been described using the pulse amplitude modulated (PAM) fluorometry technique in several studies [7,10,21,22,23,24]. The technique allows instantaneous and non-intrusive measurement of photosynthetic activity in real-time. Besides the maximum quantum yield (*F_v_/F_m_*) being used as an indicator of environmental stress [25,26], the induction of rapid light curves (RLCs) allows an evaluation of the temperature effect on photosynthetic parameters, such as photosynthetic efficiency (α), maximum electron transport rate (rETR_m_), and minimum irradiance for photosynthesis saturation (E_k_) [27]. RLC measures the effective quantum yield as a function of irradiance. An important piece of information provided by this technique is to estimate the working of a photoprotection mechanism that results in the dissipation of part of the light energy absorbed by PSII antenna in the form of heat, the non-photochemical quenching of chlorophyll fluorescence (NPQ). Furthermore, this approach makes it possible to describe the partitioning of absorbed excitation energy in PSII between three fundamental pathways, the complementary quantum yields [28]: photochemical conversion (*Y_II_*), regulated thermal energy dissipation related to NPQ (*Y_NPO_*), and non-regulated energy dissipation as heat and fluorescence, mainly due to closed PSII reaction centers (*Y_NO_*). Moreover, the influence of temperature on the photosynthetic performance of *Sargassum* was also studied by measuring dissolved oxygen concentrations in the incubation treatments [21,22,24,29,30,31]. As a general pattern, an increase in temperature inhibited photosynthesis.

In the present study (i), the thermal plume generated by the effluent from the BNPS operation was used to estimate the effect of temperature on the dynamics of the NPQ photoprotection and the effective and maximum quantum yields of PSII of Sargassum natans under field conditions, and (ii) the fate of the energy absorbed by PSII antenna was determined in *S. natans* and *Padina gymnospora*, another co-occurring brown algal species, cultured for five days at three different temperatures: 25, 31, and 34 °C, under laboratory conditions. We assessed, through the behavior of complementary quantum yields related to these temperatures, the importance of NPQ photoprotection to the survivor limits of *Sargassum* under increasing temperature.

## 2. Results

### 2.1. Assays in the Field

**Figure 1:**
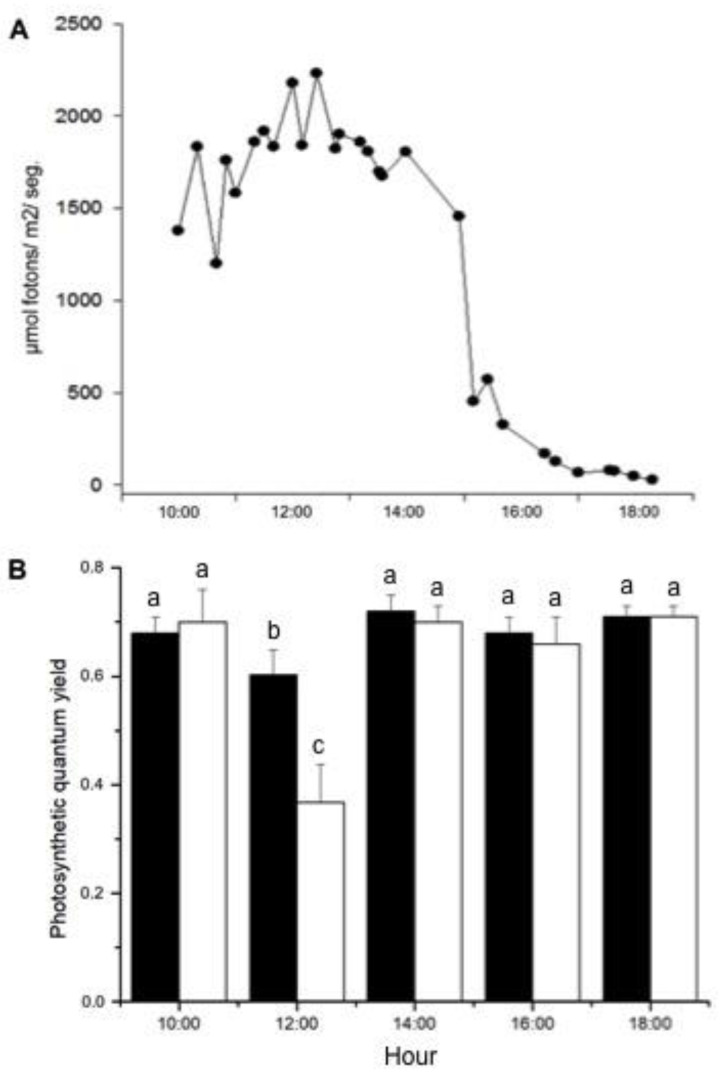
Diurnal variation of PAR and quantum yields of *Sargassum. natans,* measured on a pier located 200 m from the thermal effluent’s outfall. (**A**) PAR, plotted as 10 s-average values. (**B**) Maximum (black columns) and effective (white columns) quantum yields of plants collected at 4 m deep and transferred to the pier. Means ± SD (*n* = 8). Different letters indicate significant differences between quantum yields values (Tuckey test, *p* < 0.05).

Figure 1 shows the photosynthetic performance of *S. natans* collected from a depth outside the thermal plume at varying light intensities throughout the day. There was a significant interaction between treatments (*Φ_PSII_* and *F_v_*/*F_m_*) and measuring times (*p* < 0.001) (Supplementary Material, Table S1). During the experiment, values of the effective quantum yield (*Φ_PSII_*) were equivalent to those obtained after dark adaptation (*F_v_*/*F_m_)*, except for measurements taken at noon (ANOVA, F = 6.082, *p* = 0.005 and F = 9.499, *p* < 0.001, treatment and time, respectively). At noon, *Φ_PSII_* corresponded to 59% of the *F_v_*/*Fm* value, and *F_v_*/*F_m_* decreased by 14% compared to measurements at other times of the day. The determination of the complementary quantum yields showed that, at 12:00 h, 38% of the energy absorbed by the PSII antenna was used to perform photochemistry, whereas 33% of the energy was dissipated in a programmed form as heat (*Y_NPQ_*) and 29% of the energy was dissipated as non-programmed heat and fluorescence (*Y_NO_*).

Rapid light curves (Supplementary Material, Figure S3) provided information on the saturation characteristics of electron transport as well as the overall photosynthetic performance of *S. natans* collected at different daylight intensities as shown in Table 1. Samples collected at 10:00, 14:00, 16:00, and 18:00 h presented essentially the same photosynthetic efficiency (α), which was higher than that at noon (Supplementary Material, Table S1). The highest value of irradiance for the onset of saturation of photosynthetic activity (E_k_) was also at noon (Supplementary Material, Table S1).

**Figure 2:**
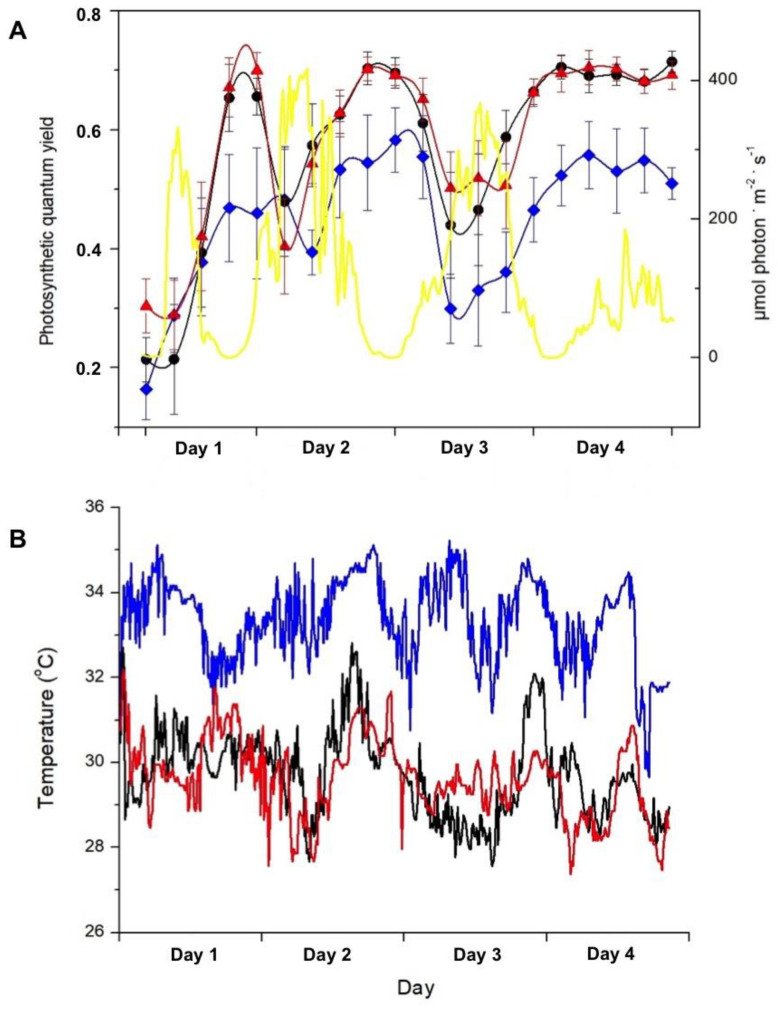
(**A**) Daily variation of PAR (yellow line) and photosynthetic quantum yield of *Sargassum natans* at 2 m deep in sites 1 (blue symbols and line), 2 (black symbols and line), and 3 (red symbols and line), respectively, 200, 500, and 1200 m away from the thermal effluent outfall, throughout four days. At each location, the effective quantum yield was determined four times a day and the maximum quantum yield was measured once each night, and PAR was continuously registered. Means ± SD (*n* = 10). (**B**) Seawater temperature at sites 1 (blue line), 2 (black line), and 3 (red line) 2 m deep, close to the studied plants, for four days.

Figure 2 shows the effect of temperature and photon flux density on the PSII quantum yield of *S. natans* measured underwater over the course of four days. Regardless of the seawater temperature, an inverse fluctuation between the quantum yield and photon flux density is observed in Figure 2A. Throughout the experiment, plants exposed to the temperature range of 32 to 35 °C (200 m distant from the thermal effluent outfall) showed a lower quantum yield than that presented by plants exposed to temperatures between 28 and 31 °C (500 and 1200 m distant from the thermal effluent outfall, as temperature ranges were essentially the same in sites 2 and 3). Compared to *S. natans* exposed to lower temperatures, plants incubated at 32–35 °C presented a decrease in the maximum quantum yield (measured at 9 p.m.) by 33% on the first day and by approximately 20% on the subsequent days. On the fourth day, the sky was overcast, and PAR values were lower than 200 µmol·m^−2^·s^−1^. Under these conditions, the quantum yield remained constant all day irrespective of temperature.

#### Underwater Net Photosynthesis and Respiration

**Figure 3:**
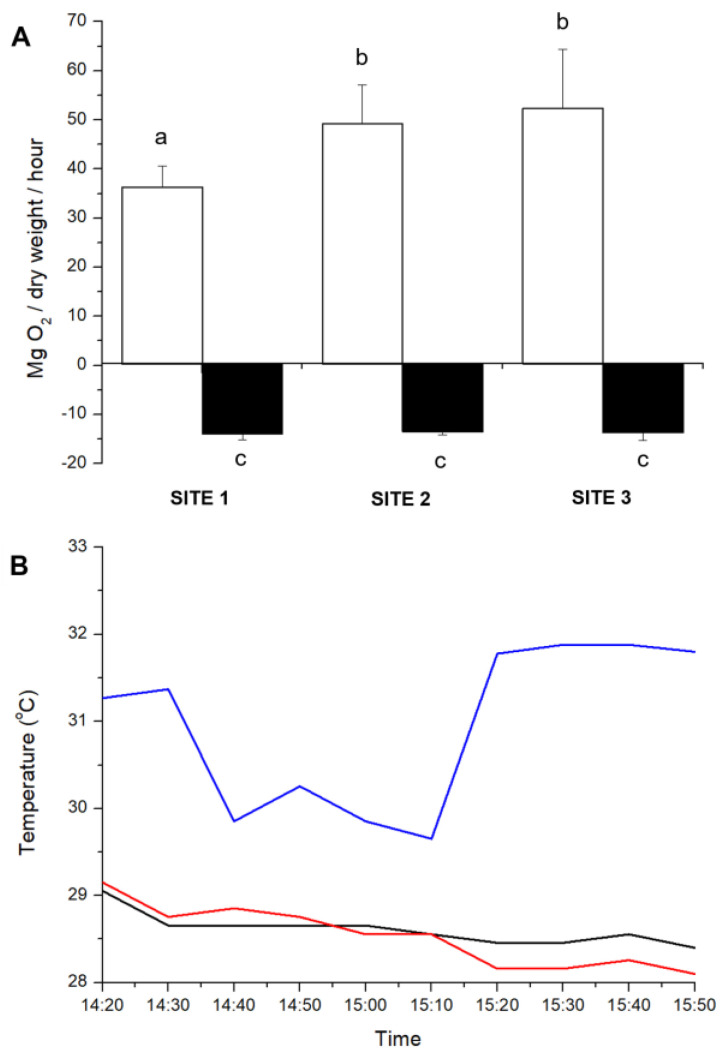
(**A**) Net oxygen production in the presence of light (white columns) and oxygen consumption in the dark (black columns) during 90 min (from 14:20 to 15:50 h) of Sargassum natans incubation in BOD bottles (2 m deep) at sites 1, 2, and 3. Means ± SD (*n* = 5). Different letters indicate significant differences. (**B**) Seawater temperature measured close to the BOD bottles at sites 1 (blue line), 2 (black line), and 3 (red line), respectively, 200, 500, and 1200 m away from the thermal effluent outfall.

Photosynthesis and respiration were differently affected by the incubation of *S. natans* for 90 min under distinct temperature ranges (Figure 3). The temperature varied between 30 and 32 °C at 200 m, and between 28 and 29 °C at 500 and 1200 m away from the thermal effluent outfall (Figure 3B). Temperatures higher than 30 °C (found at 200 m) decreased the photosynthetic activity approximately 30% in *Sargassum* (ANOVA, F = 6.34, *p* = 0.01), while respiration was not affected.

### 2.2. Assays in the Laboratory 

**Figure 4:**
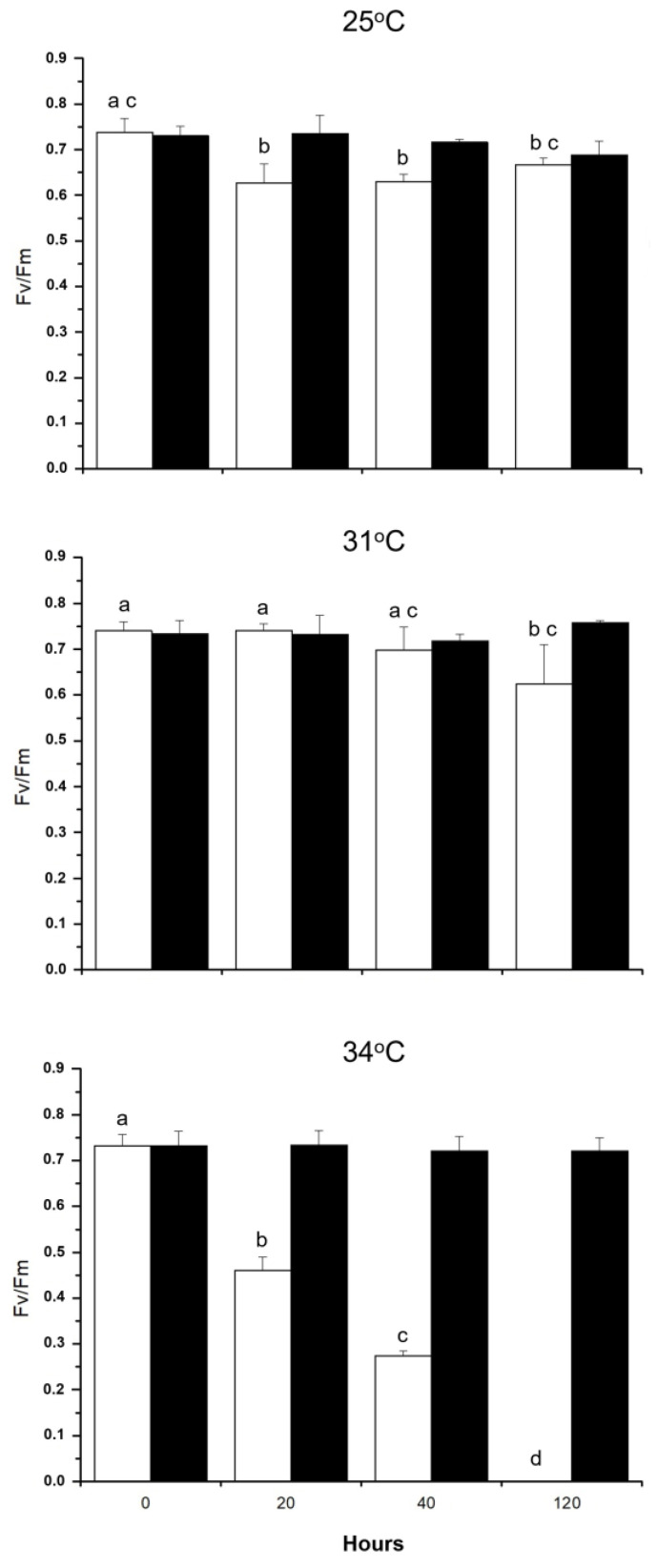
Effect of temperature on the maximum quantum yield of *Sargassum natans* (white columns) and *Padina gymnospora* (black columns). Both species were cultured in the laboratory for five days at 25, 31, and 34°C under 12 h at 90 µmol photons·m^−2^·s^−1^ irradiance. Following 20, 40, and 120 h of culturing, random plants were collected from the culture vessels, dark-adapted for 30 min for *F_v_*/*F_m_* determine. Means ± SD (*n* = 3). Different letters indicate significant differences within each temperature.

Figure 4 illustrates that increasing the cultivation temperature from 25 to 31 °C did not affect the ratio *F_v_*/*F_m_* in *S. natans*, but this parameter decreased progressively as a function of the incubation time when the temperature was 34 °C. Maximum quantum yield in *P. gymnospora* was not affected at temperatures between 25 and 34 °C (Supplementary Material, Table S2).

**Figure 5:**
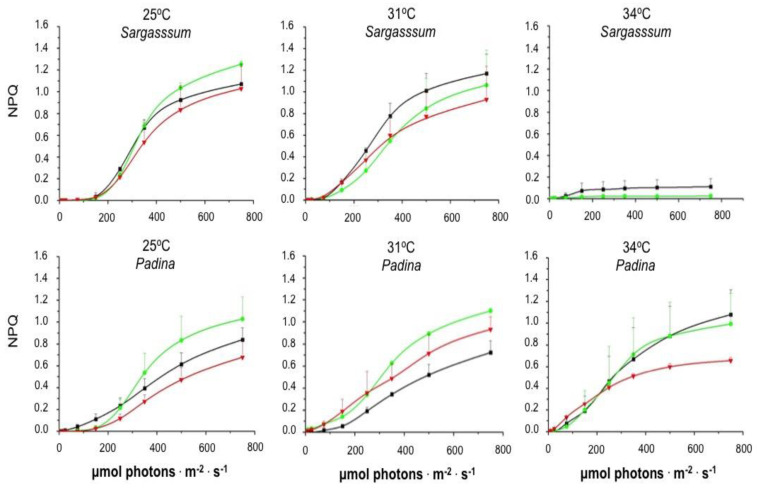
Influence of temperature on the Non-Photochemical Quenching (NPQ) as a function of photosynthetically active radiation (PAR). Both species were cultured in the laboratory at 25, 31, and 34 °C under a 12 h photoperiod and 90 µmol photons·m^−2^·s^−1^ irradiance. After 20 h (black lines), 40 h (green lines), and 120 h (red lines) at different temperatures, rapid light curves (RLCs) were obtained just after the fragments were taken from the culture flasks. Subsequently, algae were dark-adapted and *F_m_* was obtained for the calculation of NPQ as a function of increasing irradiances. Means ± SD (*n* = 3).

Figure 5 shows that the increase in PAR promoted an increase in the non-photochemical quenching parameter describing the regulated dissipation of excess energy (NPQ), regardless of the seaweed species and of the exposure time to temperatures of 25 and 31 °C. Although this same behavior is observed when *P. gymnospora* was incubated at 34 °C, NPQ development was drastically reduced and disappeared upon *S. natans* exposure to 34 °C for 20 and 40 h. We did not detect any photosynthetic response of *S. natans* cultured for 120 h at 34 °C.

**Figure 6:**
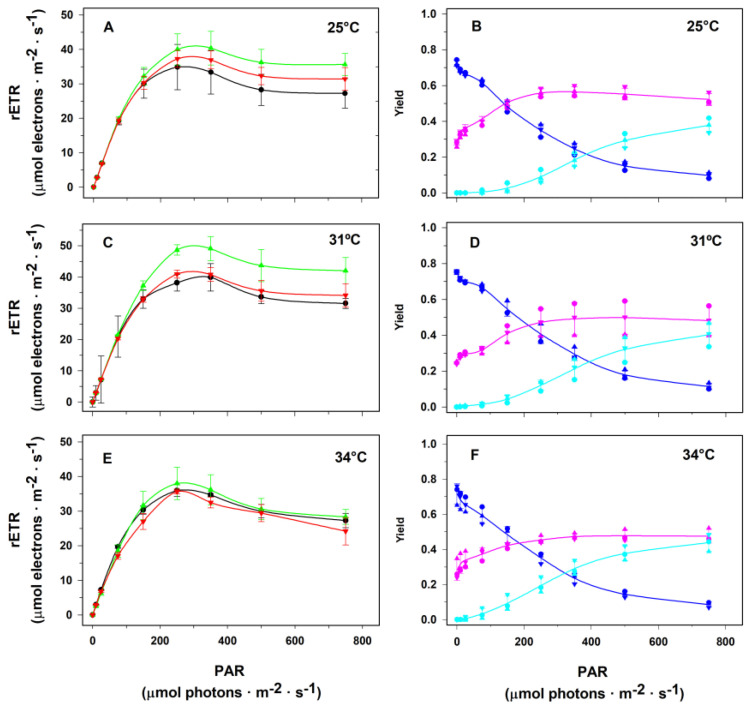
Influence of temperature and exposition time on the allocation of the energy absorbed by PSII antenna of *Padina gymnospora* cultured in the laboratory under a 12 h photoperiod at 90 µmol photons·m^−2^·s^−1^ irradiance. Rapid light curves (**A**,**C**,**E**) were obtained immediately after fragments were taken from the culture flasks after 20 h (black lines), 40 h (green lines), and 120 h (red lines) of culturing at 25 (**A**), 31 (**C**), and 34 °C (**E**). The complementary quantum yields (*Y_II_* in navy blue lines, *Y_NPQ_* in cyan lines, and *Y_NO_* in pink lines) of cultures at 25, 31, and 34 °C are presented in Panels (**B**,**D**,**F**), respectively. The complementary quantum yield lines represent average values among the three culturing times: 20, 40, and 120 h. Means ± SD (*n* = 3).

Figure 6 depicts the RLCs and the fate of excitation energy in the PSII of the macroalga *P. gymnospora* incubated for 20, 40, and 120 h at temperatures of 25, 31, and 34 °C. Regardless of the cultivation temperature, as well as the incubation time at each temperature, an increase in the relative electron transport rate (rETR) through PSII was promoted by an irradiance increase up to the range 250 to 350 µmol photons·m^−2^·s^−1^, and a decrease in rETR was observed at higher irradiances (panels 6A, 6C, and 6E). Again, irrespective of the cultivation temperature, as well as the incubation time at each temperature, we observed a decrease in the effective quantum yield with an irradiance increase presumably due to the development of the non-photochemical processes of energy dissipation, *Y_NPQ_* and *Y_NO_* (panels 6B, 6D and 6F).

**Figure 7:**
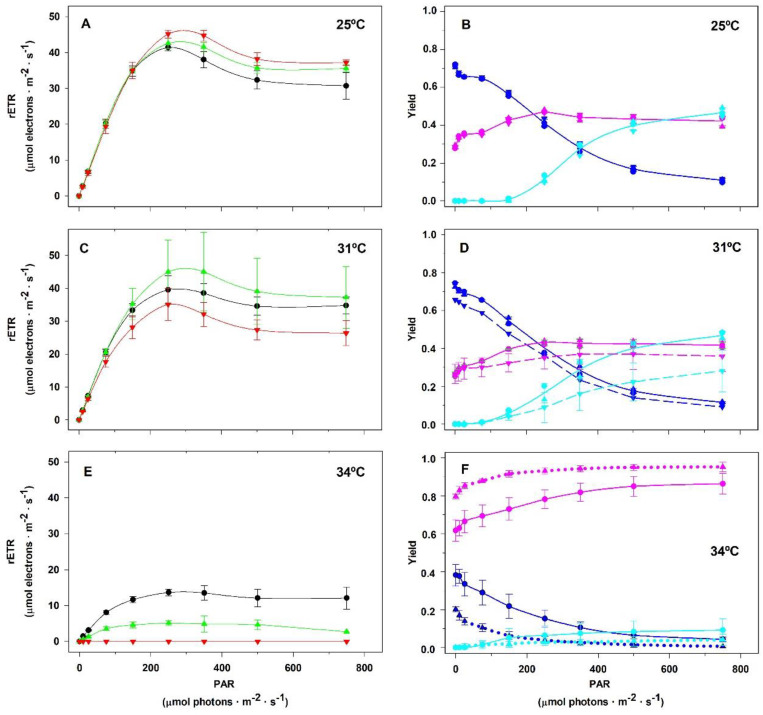
Influence of temperature on the allocation of the energy absorbed by the PSII antenna of *Sargassum natans* cultured in the laboratory at distinct temperatures under a 12 h photoperiod at 90 µmol photons·m^−2^·s^−1^ irradiance. Rapid light curves (**A**,**C**,**E**) were obtained just after the algae samples were taken from the culture vessel following 20 h (black lines), 40 h (green lines), and 120 h (red lines) of culturing at 25 (**A**), 31 (**C**), and 34 °C (**E**). The complementary quantum yields (*Y_II_* in navy blue lines, *Y_NPQ_* in cyan lines, and *Y_NO_* in pink lines) of cultures at 25 (**B**), 31 (**D**), and 34 °C (**F**) are presented in Panels (**B**,**D**,**F**), respectively. The complementary quantum yield lines in (**B**) represent average values among the three culturing times: 20, 40, and 120 h. In (**D**), the continuous lines represent average values of 20 and 40 h, whereas the dashed lines correspond to 120 h. In (**F**), continuous lines represent values of 20 h, whereas the dotted lines correspond to 40 h values. Plants incubated for 120 h at 34 °C did not show any photosynthetic activity. Means ± SD (*n* = 3).

Figure 7 reveals that *S. natans* is sensitive to temperatures above 31 °C. Panel 7A shows that the incubation time at 25 °C did not affect the linear region of the RLCs and that the maximum rETR was reached between 250 and 350 µmol photons·m^−2^·s^−1^, as previously observed in *P. gymnospora*. However, the incubation for 120 h at 31 °C resulted in a decrease in the initial slope of the RLC compared to those obtained after incubation for 20 and 40 h at 31 °C (panel 7C). Exposure of *S. natans* for 20 h at 34 °C resulted in a marked decrease in the rate of electron transport through the PSII promoted by the actinic radiation (panel 7E). Together with panel 7F, we verify that increasing the incubation time to 40 h at 34 °C resulted in the almost complete inhibition of the fraction of energy that is photochemically converted into the PSII reaction center (*Φ_PII_*). Under these conditions, *Y_II_* approached zero and no energy was dissipated as heat through the NPQ-regulated photoprotective mechanism (*Y_NPQ_*). At the same time, we observe a sharp increase in the *Y_NO_* values that could reflect the inability of *S. natans* to protect itself against damage from excessive light. Samples submitted to 120 h at 34 °C did not have any photosynthetic activity.

**Figure 8:**
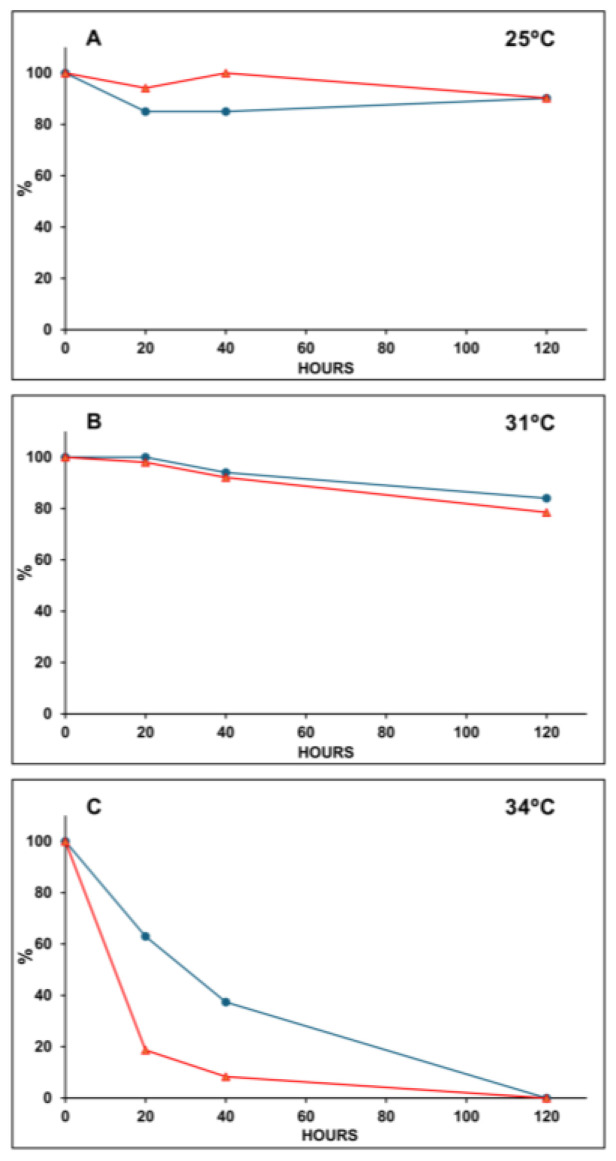
Effects of the temperature (25, 31, and 34 °C) on the maximum quantum yield, *F_v_/F_m_* (blue line), and the quantum yield of regulated non-photochemical energy loss, *Y_NPQ_* (red line), in *Sargassum natans* (Panels (**A**–**C**)). The *F_v_/F_m_* data were taken from the values represented in Figure 4 for S. natans, and the percentage values are relative to *F_v_/F_m_* measurements at time zero (100%) at each temperature. The *Y_NPQ_* data were taken from the values represented in Figure 7 for *S. natans* under irradiance of 750 µmol photons·m^−2^·s^−1^. The percentage values were calculated from the highest *Y_NPQ_* value at 25 °C (chosen because it is the temperature that maintained the photosynthetic parameters at their best), which was considered 100% as time zero.

Figure 8C shows that the exposure of *S. natans* to 34 °C for 20 h led to a decrease in the regulated dissipation of the energy absorbed by the PSII antenna (*Y_NPQ_*) of approximately 81%, while *F_v_/F_m_* declined by only 37%, revealing that *Y_NPQ_* fell approximately 2 times faster than *F_v_/F_m_*. Under these same conditions, exposure for 40 h reduced *Y_NPQ_* by approximately 92%, while *F_v_/F_m_* decreased by 63%. Indeed, the deleterious effect of temperature on these quantum yields can be observed in Panel B of Figure 8, since the exposure of S. natans to 31 °C for 120 h decreased *Y_NPQ_* and *F_v_/F_m_* by 21.5 and 16%, respectively. It is worth noting that the quantum yields in *P. gymnospora* were not affected at temperatures in the range of 25–34 °C. Complete data are presented in Table S3 of Supplementary Material.

## 3. Discussion

Our results clearly show that the photosynthetic activity of *S. natans* was significantly affected by temperature. Net photosynthetic rates were decreased in algae incubated in the vicinity of the warm water effluent (200 m distance). Likewise, the effective quantum yield was also negatively affected by high temperatures. When manipulating the exposure of *S. natans* to increased temperatures by incubating plants at closer distances to the thermal effluent outfall, an inverse relation between PAR and the photosynthetic quantum yield of PSII was observed, regardless of temperature. Over the four-day incubation, the quantum efficiency of PSII was maximal in the absence of light (21:00 h), decreased to a minimum towards midday, and recovered in late afternoon to the values recorded in the early morning during the diurnal light cycle. Again, NPQ and *Φ_PSII_* were inversely related, where *Φ_PSII_* decreased with increasing irradiance as more electrons could accumulate at the PSII acceptor side. Then, there was a relative increase in NPQ of the energy absorbed by the PSII antenna in the form of thermal energy dissipation [26]. Because of the reduction in the incident light on the fourth day, no dissipation of energy occurred under the two temperature ranges and the quantum yields remained constant all day. It is noteworthy that at a temperature range of 28–31 °C (500 and 1200 m from the thermal effluent outfall), *Sargassum* showed a substantial difference in *F_v_/F_m_* (measured at 21:00 h) compared to exposure to higher temperatures (32–35 °C). The stress induced by incubation at 32–35 °C caused a decrease in *F_v_/F_m_* by 33% on the first day and approximately 20% on the subsequent days. At the beginning of the experiment, regardless of the temperature, quantum yields were very low in the absence of light, probably indicating some sort of stress caused by the transplantation of the macroalgae.

As described for other *Sargassum* species [7,21,32], *S. natans* collected from outside the thermal plume (at 4 m deep and at a temperature of 22 °C over the course of the day) exhibited the characteristics of a sun-adapted plant, as evidenced by the daily variation in *F_v_/F_m_* and *Φ_PSII_*, suggesting the occurrence of dynamic photoinhibition. The initial value of *F_v_/F_m_* in the morning was about 0.7, which is comparable to the levels observed for other *Sargassum* species [7,22,23,32]. Photosynthesis, measured as *F_v_/F_m_* and *Φ_PSII_*, decreased at noon and then recovered rapidly to the values recorded in the morning, probably due to the combined factors of decreased irradiance and an increase in the water column due to the tidal variation between noon and 14:00 h. Over this period time, there was an increase of approximately, 0.6 m in the water column (Brazilian Navy’s Board of Hydrography and Navigation). The rapid recovery of *Φ_PSII_* suggests that the responses of *S. natans* to high light intensity are linked to dynamic photoinhibition, i.e., a photoprotective mechanism that involves a reversible down-regulation of the PSII activity. Thus, at midday, light-adapted *Φ_PSII_* was 41% lower than the one measured for dark-adapted plants (*F_v_*/*F_m_*), due to the inherent impact of non-photochemical quenching (NPQ) reducing the light-adapted quantum yield [33]. These results were reinforced by the photosynthetic performance parameters (rETR_max_, alpha, and E_k_) obtained from the RLC performed at different times of day (from 10:00 to 18:00 h). Whereas the rETR_max_ essentially presented the same value, the decrease in alpha and the increase in E_k_ support the downregulation of the photosynthetic activity of *S. natans* at midday. It has been reported that an increase in NPQ can reduce the alpha value and avoid damage from excess light reaching PSII [23,34].

High temperature severely inhibited the photosynthetic performance of *S. natans* the under controlled conditions of the laboratory. The use of *F_v_/F_m_* as an indicator of stress revealed the thermolability of the photosynthetic apparatus of *S. natans* at 34 °C since incubation for 20 h resulted in a 37% decrease in *F_v_/F_m_* and a decline of 62.6% after incubation for 40 h. No activity was detected following 120 h incubation at 34 °C. By contrast, the photosynthetic activity of *P. gymnospora* was not affected by cultivation at 34 °C, indicating that the plants of *P. gymnospora* growing in IGB are adapted or acclimated to elevated temperatures, being tolerant to temperatures higher than 30 °C, as suggested by Széchy et al. [13].

In algae, high temperature reduces or even inactivates PSII, Rubisco, and Rubisco activase, severely inhibiting photosynthesis [17,18,19]. Thus, the decline in carbon fixation via the Calvin cycle decreases the demand for reducing equivalents from the photosynthetic electron transport chain and the absorption of excitation energy exceeds the capacity for its utilization, causing photodamage to the photosynthetic apparatus. In these events, massive production of reactive oxygen species (ROS) causes oxidative stress that destroys cell membrane structures and leads to disorders of intracellular metabolism [35,36]. One of the defense mechanisms underlying the oxidative stress caused by high temperatures consists of a system that includes antioxidant enzymes, such as superoxide dismutase and peroxidases, which can mitigate oxidative damage by removing the ROS formed under high-temperature stress [20,24]. Moreover, a multitude of photoprotection mechanisms were selected throughout evolution in plants and algae for their role in preventing damage by the action of ROS. One of the most important protection mechanisms is the dissipation of excessive excitation energy as heat in the light-harvesting complexes of the photosystems, termed NPQ [35].

NPQ is often regarded as an indicator to a physiological stress [37], which has been proved in studies on algae [16,38], including *Sargassum natans* [39]. This process is triggered by the formation of a pH gradient (ΔpH) across the thylakoid membrane. The resulting acidification of the lumen promotes the protonation of specific proteins of PSII antenna, which causes a rapid induction of NPQ [40]. Lumen acidification also activates de-epoxidase enzymes involved in a light-dependent carotenoid interconversion, the xanthophyll cycle, which plays a key role in the on- and offset of NPQ. In contrast to higher plants, the trans-thylakoid proton gradient alone does not induce NPQ in brown algae, which is only established when the light-driven pH is accompanied by the accumulation of the carotenoid zeaxanthin [41,42,43]. The general importance of NPQ for the photoprotection of plants and algae is documented by its wide distribution among photosynthetic organisms.

We observed that NPQ induction, by increasing irradiance, showed a sigmoidal-shaped NPQ pattern in the two brown algae cultured in the laboratory. Neither the induction pattern nor the NPQ capacity was affected by the exposure time (20, 40, and 120 h) to temperatures of 25, 31, and 34 °C, except for *S. natans* exposed to 34 °C. In this case, a virtually total inhibition of NPQ occurrence was observed after incubation for 20 h at 34 °C. It is worth noting that the photoprotection (NPQ) mechanism to prevent damage to the photosynthetic apparatus was completely inhibited by high temperature before the complete decrease in *F_v_*/*F_m_*, indicating that the loss of photoprotection preceded photodamage. Thus, the antioxidant enzymes responsible for ROS scavenging [20,24,43,44] are likely unable to cope with the massive increase in ROS caused by the high-temperature-induced NPQ inhibition.

After 120 h of *S. natans* cultivation under non-stressful conditions (25 °C, 12 h photoperiod, and 90 μmol photon m^−2^ s^−1^ PAR), we found that circa 12, 44, and 44% of the absorbed energy from 750 μmol photon m^−2^ s^−1^ PAR were allocated, respectively, among *Y_II_*, *Y_NPQ_*, and *Y_NO_*. Under the same conditions, we found that *P. gymnospora* shared 10, 34, and 56% of the energy absorbed by the PSII, respectively, among *Y_II_*, *Y_NPQ_*, and *Y_NO_*. Similar figures were found when *P. gymnospora* was cultivated for 40 h at 34 °C, whereas the fate of excitation energy in PSII among *Y_II_*, *Y_NPQ_*, and *Y_NO_* in *S. natans* corresponded, respectively, to 1, 4, and 95%. According to Wang et al. [45], a high *Y_NPQ_* indicates that the photon flux density is excessive and that the plant sample was able to protect itself by the dissipation of excessive excitation energy into harmless heat. Without such dissipation, there would be the formation of ROS, which causes irreversible damage. In contrast, high *Y_NO_* indicates that both photochemical and regulated non-photochemical capacities are inefficient.

Field results reinforce that the photosynthesis of benthic *S. natans* is highly sensitive to high-temperature stress and, in agreement with previous studies [21,22,24,29,30,31], is inhibited before dark respiration is affected. In fact, photosynthesis in algae is extremely sensitive to high-temperature stress, as it can induce PSII inactivation, and destroy algal membranes and thylakoids, thereby inhibiting photosystem activities [19]. Moreover, high temperatures reduce the activity of Rubisco, limiting photosynthetic carbon assimilation [18,46].

The effect of rising sea surface temperatures on habitat-forming algae is critical to predict their responses to global warming, specifically to the increasing frequency of heat waves [47]. Better approaches to conserve marine forests against climate change and anthropogenic disturbances should be designed based on increasingly robust species distribution models [48], which should aggregate multiple biological information, from different species and populations from different regions. Specifically, for IGB, the influence of other environmental factors, such as salinity, the concentration of nutrients and pollutants in the seawater, and interaction with biotic factors, such as herbivory and competition, need to be studied in order to guide and strengthen protection initiatives at its *Sargassum* beds. The integrative nature of such models should be sufficiently informative to provide successful restoration measures for these habitat-forming species [49], and simultaneously their conservation.

## 4. Materials and Methods

### 4.1. Biological Material

Adult plants of the brown algae Sargassum natans (Linnaeus), Gaillon (Ochrophyta, Fucales), and *Padina gymnospora* (Kützing) Sonder (Ochrophyta, Dictyotales) were collected from the benthic populations of Ilha Grande Bay (IGB), Rio de Janeiro State, Brazil: a warm temperate Southwestern Atlantic region [50]. The plants identified as *S. natans* followed the phylogenetic concept of species based on three genetic markers (See Wynne [51]), that grouped plants with great morphological variability.

### 4.2. Field Studies

Field studies were performed in Ilha Grande Bay (IGB), Rio de Janeiro State, Brazil: a warm temperate Southwestern Atlantic region [32]. Inside IGB, the sole Brazilian Nuclear Power Station (BNPS) has operated since the 1980s and continuously discharges its heated effluent into the inner part of Piraquara de Fora Cove, where it forms a thermal plume [12,52]. It was considered that the temperature gradient of the surface seawater within Piraquara de Fora Cove constitutes an adequate condition to test the influence of increased temperatures on the photosynthetic performance of *S. natans* in the natural environment. In addition, there is a great extension of unconsolidated sandy bottom at a depth of 2 m with sites sheltered from the swells of the south and north quadrants, with little exposure to wave action.

#### 4.2.1. Measurements on a Pier

Plants of *S. natans* were collected (*n* = 8) by the autonomous diving at 200 m from the thermal effluent’s outfall (23°00′47.47″ S and 44°26′40.92″ W), below the thermal plume (around 22 °C) and around 4 m deep, and brought to the surface. On a pier, the photosynthetic active radiation (PAR), maximum and the effective quantum yields were measured between 10:00 and 18:00 h for describing the diurnal variation.

#### 4.2.2. Underwater Measurements

The photosynthetic quantum efficiency of *S. natans* was also determined underwater during a four-day experiment under different temperature ranges. For using the thermal plume as a temperature gradient, we chose three sites located at 200 m, 500 m, and 1200 m (sites 1, 2, and 3, respectively), away from the thermal effluent outfall. During the experiment, site 1 presented temperatures from 31 to 35 °C whereas sites 2 and 3 presented temperatures from 28 to 32 °C. Adult plants still attached to boulders were collected by autonomous diving around 2 m deep at Sabacu Island (23°00′25.51″ S and 44°23′3.58″ W), located outside the thermal plume. The boulders with plants, kept inside a shaded container with local seawater at around 27 °C, were transported by speedboat to a site inside Piraquara de Fora Cove. Plants were cleaned from macroscopic epiphytes and were then positioned 2 m deep near site 3 for overnight acclimation. On the next day, boulders with 10 plants, haphazardly selected, were transferred to sites 1, 2, and 3 at 2 m deep summing 10 plants per site (Supplementary Material, Figure S1). At each site, the effective quantum yield of the plants of the transplanted boulders was determined underwater four times a day (10:00 h ± 20 min, 12:00 h ± 20 min, 14:00 h ± 20 min, and 16:40 ± 20 min) and the maximum quantum yield was measured once each night (between 20:00 and 21:00 h).

### 4.3. Laboratory Assays

Twenty adult plants of *S. natans* and *P. gymnospora* were collected by snorkeling in the shallow subtidal rocky bottom (1.0 m deep), outside Piraquara de Fora Cove (22°59′38.0″ S and 44°25′53.6″ W), SST around 28 °C. The plants were cleaned of epiphytes, rinsed in clean seawater, and transported to the laboratory in coolers.

Apical fragments around 5 cm long of *S. natans* and *P. gymnospora* were left in culture chambers to acclimate (12 h photoperiod, 90 μmol photon m^−2^ s^−1^) in conical flasks filled with 2000 mL of Provasoli-enriched seawater (1 mL/L) and constant aeration at 25 °C. After 48 h of acclimation, the fragments were transferred to a new culture medium and the experiment was set at three different temperatures: 25, 31, and 34 °C, maintaining the other culture conditions. The photosynthetic performance of three specimens of *S. natans* and three of *P. gymnospora* were measured at the beginning of the experiments and after 20, 40, and 120 h of incubation.

### 4.4. Photosynthesis Measurements

#### 4.4.1. Chlorophyll a Fluorescence

Fluorescence induction was measured using a diving-PAM system (Walz GmbH, Germany), equipped with a blue LED (470 nm) and an 8 mm diameter standard glass-fiber optic probe (Walz, Effeltricht, Germany), placed 10 mm away from the thalli. An internal halogen lamp provided the actinic illumination for each given irradiance level, as well as the saturating pulses. The Diving-PAM settings were as follows: measuring light intensity 2, saturating light intensity 8, saturating width 0.8 s, gain 2, and damping 2.

The effective quantum yield of PSII, *Φ_PSII_* = (*F’_m_ − F_s_)/F’_m_*, was determined in light-adapted samples at a particular irradiance level, where Fs is the steady-state fluorescence level and *F’_m_* is the maximal fluorescence yield induced by a saturating light pulse (6000 µmol photons·m^−2^·s^−1^ for 800 ms). To determine the maximum quantum yield of PSII, plants were dark adapted for 30 min before measurements. Thereafter, the minimum fluorescence level (*F_o_*) was detected under the modulated measuring light of the PAM (a weak pulsed light; < 1 μmol photons m^−2^·s^−1^), whereas the maximum fluorescence level (*F_m_*) was obtained by exposing the plants to a pulse of saturating light (6000 µmol photons·m^−2^s^−1^ for 800 ms) in the presence of modulated light. Variable fluorescence (*F_v_*) was calculated from *F_m_-F_o_*, and the maximum quantum efficiency of PSII photochemistry was obtained from the ratio *F_v_/F_m_*, which expresses the maximum quantum efficiency of primary photochemistry [26]. The non-photochemical quenching (NPQ = (*F_m_ − F’_m_) /F’*_m_) reflects energy dissipated as heat-related via the energization of thylakoid membranes due to lumen acidification and light-induced xanthophyll cycle operation [53].

Rapid light curves (RLCs) were obtained by exposing the plants to eight predefined incremental light intensities for 10 s at each level of irradiance [27], using the software “Wincontrol-3” (Walz GmbH). Different PAR levels used to obtain RLCs were defined using the micro-quantum sensor (Walz GmbH, Effeltrich, Germany) calibrated against a Li-Cor quantum sensor (Li-Cor, Lincoln, NE, USA). The relative Electron Transport Rate (rETR) was obtained by multiplying *Φ_PSII_* by the density value of PAR of each light intensity [33], and rETR values were fitted to the RLC according to Platt et al. [54].

The fate of the absorbed energy by PSII antenna between photochemical dissipation (*Y_II_*), regulated non-photochemical dissipation (*Y_NPQ_*), and non-regulated non-photochemical dissipation passively lost as heat and fluorescence (*Y_NO_*) was estimated by determining the complementary PSII quantum yields [28] as follows:

*Y_II_* = *(F’_m_ − F_s_)/F’_m_* (quantum yield of photochemical energy conversion)

*Y_NPQ_* = (*F/F’_m_) − (F/F_m_*) (quantum yield of regulated non-photochemical energy loss)

*Y_NO_* = F/F_m_ (quantum yield of non-regulated non-photochemical energy loss)

*Y_II_* + *Y_NPQ_* + *Y_NO_* = 1

#### 4.4.2. Net Photosynthesis and Respiration

Net photosynthesis and respiration estimates were obtained by measuring dissolved oxygen according to the modified Winkler’s method [55] in BOD bottles (300 mL), under light and dark (wrapped in aluminum foil) conditions, each one containing a 5 cm apical branch of adult plants of *S. natans.* Adult plants and seawater were collected 2 m deep at Sabacu Island by autonomous diving. The seawater used in the experiment was filtered by 45 µm pores (Millipore^®^). Membranes with pores of 45 microns do not remove microalgae and, probably, seawater filtered in this way may contain some phytoplankton. Although the literature shows that increasing ambient temperature increases respiratory activity, this did not occur in our case, since oxygen consumption remained unchanged. Therefore, if phytoplankton was present, its influence on oxygen consumption would be negligible. In addition, microalgal photosynthesis is increased by increasing temperature up to 30 °C. Thus, if present, microalgae would mitigate the observed decline in oxygen production by Sargassum when the temperature increased from 25 to 31 °C.

One-meter-long PVC tubes were used as a support to anchor the BOD bottles (Supplementary Material, Figure S2). These experimental modules were attached to a ballast and a buoy by a rope to maintain the incubation 2 m deep at sites 1, 2, and 3. After the incubation of the BOD bottles for 90 min in light and dark conditions, dissolved oxygen was fixed in 3 M manganese chloride solution and alkaline iodine solution (NaOH 8 M, NaI 4 M) and kept cool in the dark. Twenty-four hours after oxygen fixation, 80 µL of 10 M sulfuric acid was added and absorbances were spectrophotometrically determined at 466 nm (Kasvi, model K37-UV-VIS 190–1100 nm). *S. natans* fragments were oven-dried at 60 °C until they reached constant weight. The results were expressed in milligrams of O_2_ per dry weight in grams and the incubation time in hours.

### 4.5. Abiotic Measurements

When the photosynthetic performance was determined in plants brought to the surface, PAR was measured as 10 s-averaged values using a portable light meter LI-250A with a spherical quantum sensor LI-193SA (Li-Cor Inc., Lincoln, NE, USA). During the underwater experiment, PAR was measured 2 m deep every 10 min for the four-day experiment using a PAR logger (Odyssey, Christchurch, New Zealand). The PAR sensor was attached to a ballast and kept close to the transplanted fragments of *S. natans*. In the laboratory experiments, PAR was measured as 10 s averaged values using a portable light meter LI-250A with a flat quantum sensor LI-190SA, cosine-corrected up to 80° angle of incidence (Li-Cor Inc., USA) on the outer surface of the culture flasks.

In the field, seawater temperature was measured with a Hobo Pendant temperature logger (Onset, Cape Cod, MA, USA). In the laboratory, temperature was measured with a mercury thermometer.

### 4.6. Statistical Analyses

One-way and two-way analyses of variance (ANOVA) were applied for the comparisons among the treatments of each assay, followed by a Tukey test, considering *p* < 0.05 significant values. More information regarding each test is given in the legends of figures and tables. Shapiro–Wilk and Levene tests were used for assessing departures from normality and homogeneity of variances, respectively. Data were not transformed for the analyses. The R-environment [56] was performed; particularly for the pairwise comparisons, and the Multcomp Package was used [57].

## 5. Conclusions

*Sargassum natans* and *Padina gymnospera*, from a warm temperate region and growing in similar environments, have different responses to increased seawater temperatures. *S. natans* showed a clear dynamic photoinhibition indicated by the daily variation of the efficiency of quantum yields. It also indicated that the stress induced by increasing temperature to the range of 32–35°C over four days in the field caused a decrease in the photosynthetic capacity. In the laboratory, the effect of increasing exposure to higher temperatures points to a lower tolerance of *S. natans* above 31°C, indicated by the substantial drop in the rate of electron transport. Unlike *S. natans*, *P. gymnospora* remained with high values of the same fluorescence parameters even at 34°C, indicating to be better equipped to thrive at temperatures higher than those presently occurring. Another result to be highlighted is a stronger decline of the regulated non-photochemical quenching of chlorophyll fluorescence (*Y_NPQ_*), when compared to the decline of maximum quantum yield of PSII. It seems to indicate that PSII is rapidly destroyed in the absence of this photoprotection. Based on this study, it is predicted that heatwaves might suppress the growth of this species from shallower waters. Temperature increase beyond 32 °C might jeopardize *Sargassum* populations in nature. Our results can subside conservationist strategies and prediction models for population behavior in response to climate changes. Further studies including the relationship of *Y_NPQ_* and oxidative stress would greatly improve the understanding of this scenario and improve our prediction power.

## Figures and Tables

**Table 1:** Diurnal variation in the maximum relative electron transport rate (rETR_max_), the photosynthetic efficiency (α), and the highest value of irradiance to begin the saturation of photosynthetic activity (E_k_) of *Sargassum natans* collected at 4 m deep and transferred to a pier at 10:00, 12:00, 14:00, 16:00, and 18:00 o’clock. The times correspond to GMT-3 (Greenwich Mean Time). Means ± SD (*n* = 5).

Time (h)	rETR_max_ (µmol Electrons·m^−2^·s^−1^)	α (µmol Electrons)/(µmol Photons)	E_k_ (µmol Photons·m^−2^·s^−1^)
10:00	19.4 ± 2.0	0.384 ± 0.035	51 ± 4
12:00	17.8 ± 1.5	0.227 ± 0.048	62 ± 5
14:00	17.8 ± 1.6	0.422 ± 0.012	42 ± 3
16:00	16.6 ± 1.4	0.411 ± 0.019	40 ± 4
18:00	16.1 ± 3.3	0.409 ± 0.043	32 ± 2

## Data Availability

The raw data supporting the conclusions of this article will be made available by the authors on request.

## Supplementary Materials

The following supporting information can be downloaded at https://www.mdpi.com/article/10.3390/plants14050759/s1, Figure S1: Underwater chlorophyll a fluorescence measurements; Figure S2: net oxygen production in BOD bottles; Figure S3: Diurnal variation of Rapid Light Curves of *Sargassum natans;* Table S1: Statistical analyses of the photosynthetic parameters of *Sargassum natans* between the different times of day; Table S2: Statistical analyses of maximum quantum yield of algae cultured in laboratory; Table S3: Comparison of temporal effects of temperature on *Fv/Fm* and *Y_NPQ._*
