# Supplementary material for: Response of a Benthic Sargassum Population to Increased Temperatures: Decline in Non-Photochemical Quenching of Chlorophyll a Fluorescence (NPQ) Precedes That of Maximum Quantum Yield of PSII
Source: Plants (Basel). 2025 Mar 1;14(5):759. doi: 10.3390/plants14050759 (PMC11901439; doi:10.3390/plants14050759)
Supplement: Supplementary file 1 [file plants-14-00759-s001.zip › PLANTS Supplementary Material, Figure S2.pdf]

# Response of a Benthic *Sargassum* Population to Increased Temperatures: Decline of Non-Photochemical Quenching of Chlorophyll a Fluorescence (NPQ) Precedes That of Maximum Quantum Yield of PSII

## Supplementary Material

### Figure S2

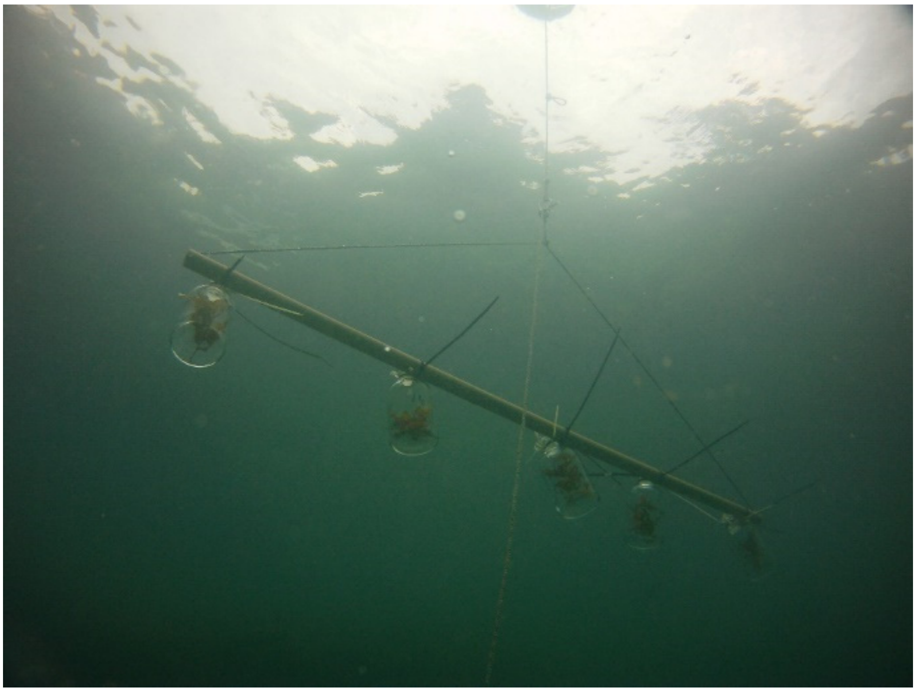

Incubation of *Sargassum natans* in DBO bottles 2 m deep for measuring net oxygen production. Incubation time for 90 min on a clear day.
