# Supplementary material for: Response of a Benthic Sargassum Population to Increased Temperatures: Decline in Non-Photochemical Quenching of Chlorophyll a Fluorescence (NPQ) Precedes That of Maximum Quantum Yield of PSII
Source: Plants (Basel). 2025 Mar 1;14(5):759. doi: 10.3390/plants14050759 (PMC11901439; doi:10.3390/plants14050759)
Supplement: Supplementary file 1 [file plants-14-00759-s001.zip › PLANTS Supplementary Material, Figure S3.pdf]

Response of a Benthic *Sargassum* Population to Increased Temperatures: Decline of Non-Photochemical Quenching of Chlorophyll a Fluorescence (NPQ) Precedes That of Maximum Quantum Yield of PSII

Supplementary Material

Figure S3

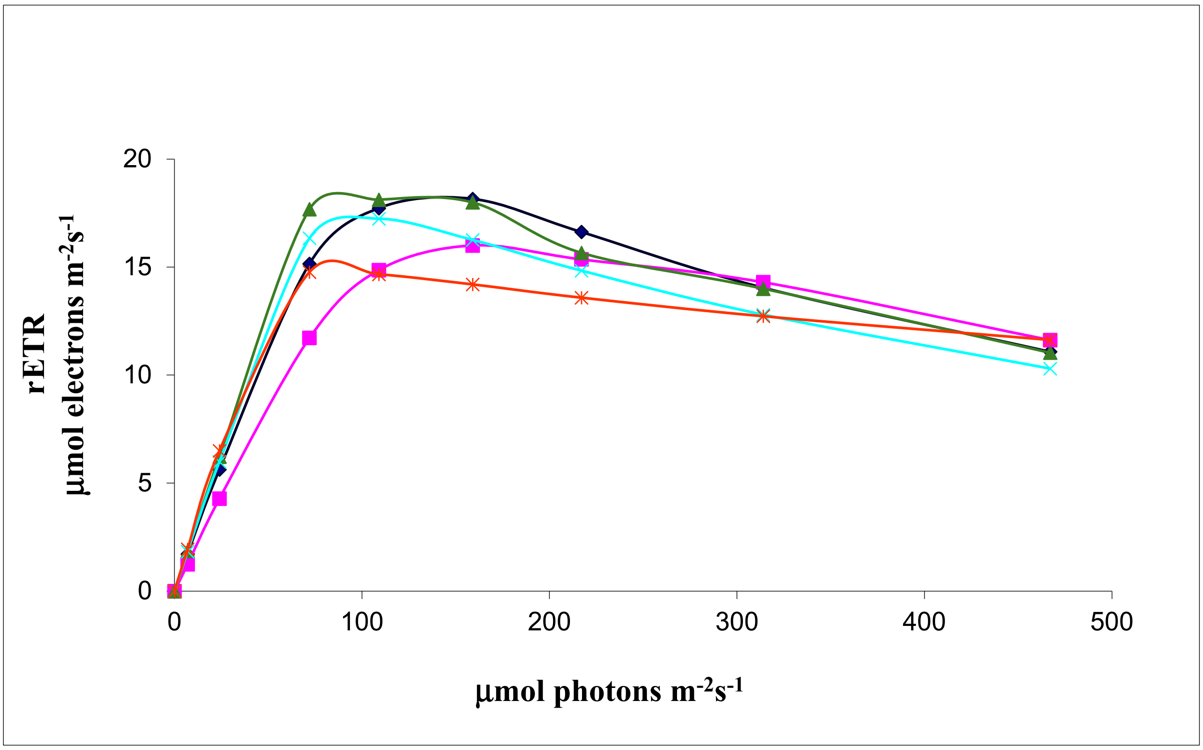

Rapid Light Curves of *Sargassum natans* collected by autonomous diving 200 m from the thermal effluent outfall, around 4 m deep and below the thermal plume. Relative rates of electron transport through PSII (rETR) were obtained by exposing the plants to different light intensities for 10 s at each irradiance level (between 0 and 500 µmol photons m^{-2} s^{-1}). Measurements were made at 10:00 (black line), 12:00 (pink line), 14:00 (green line), 16:00 (blue line), and 18:00 o'clock (red line).Values are means (n=8) and SD are not represented for better observation.
