# Supplementary material for: Response of a Benthic Sargassum Population to Increased Temperatures: Decline in Non-Photochemical Quenching of Chlorophyll a Fluorescence (NPQ) Precedes That of Maximum Quantum Yield of PSII
Source: Plants (Basel). 2025 Mar 1;14(5):759. doi: 10.3390/plants14050759 (PMC11901439; doi:10.3390/plants14050759)
Supplement: Supplementary file 1 [file plants-14-00759-s001.zip › PLANTS Supplementary Material, Table S1.pdf]

Response of a Benthic *Sargassum* Population to Increased Temperatures: Decline of Non-Photochemical Quenching of Chlorophyll a Fluorescence (NPQ) Precedes That of Maximum Quantum Yield of PSII

SUPPLEMENTARY MATERIAL

Table S1

Comparison of different photosynthetic performance parameters (F_v/F_m, Φ_{PSII}, $rETR_{max}$, and E_k) of adult plants of *Sargassum natans* among different times of the day (10:00, 12:00, 14:00, 16:00, and 18:00 h), presented in **Figure 1** and **Table 1**. Tukey Test: significant differences between hours in bold, p<0.05. df= degrees of freedom.

Contrast (hours)	t-ratio	p-value
F_v/F_m (df=35)		
10 x 12	5.188	**0.0001**
10 x 14	-2.097	0.2441
10 x 16	-0.326	0.9974
10 x 18	-1.701	0.4466
12 x 14	-7.285	**<0.0001**
12 x 16	-5.514	**<0.0001**
12 x 18	-6.889	**<0.0001**
14 x 16	1.771	0.4061
14 x 18	0.396	0.9946
16 x 18	-1.375	0.6474
Φ_{PSII} (df= 25)		
10 x 12	11.744	**<0.0001**
10 x 14	-0.814	0.9239
10 x 16	0.736	0.9458
10 x 18	-0.950	0.8746
12 x 14	-12.558	**<0.0001**
12 x 16	-11.007	**<0.0001**
12 x 18	-12.693	**<0.0001**
14 x 16	1.550	0.5412

14 x 18	-0.136	0.9999
16 x 18	-1.686	0.4601
Alpha (df= 20)		
10 x 12	6.846	**<0.0001**
10 x 14	-1.991	0.3057
10 x 16	-1.582	0.5250
10 x 18	-0.800	0.9275
12 x 14	-8.837	**<0.0001**
12 x 16	-8.427	**<0.0001**
12 x 18	-7.646	**<0.0001**
14 x 16	0.409	0.9936
14 x 18	1.191	0.7564
16 x 18	0.782	0.9329
$rETR_{max}$ (df= 20)		
10 x 12	0.833	0.9171
10 x 14	0.843	0.9137
10 x 16	1.873	0.3627
10 x 18	1.412	0.6272
12 x 14	0.010	1.000
12 x 16	1.040	0.8340
12 x 18	0.579	0.9767
14 x 16	1.030	0.8387
14 x 18	0.569	0.9781
16 x 18	-0.461	0.9900
E_k (df= 20)		
10 x 12	-4.703	**0.0012**
10 x 14	3.182	**0.0338**
10 x 16	4.106	**0.0044**
10 x 18	7.107	**<0.0001**
12 x 14	7.885	**<0.0001**
12 x 16	8.809	**<0.0001**
12 x 18	11.810	**<0.0001**
14 x 16	0.924	0.8841
14 x 18	3.925	**0.0067**
16 x 18	3.001	**0.0491**
