# Supplementary material for: Response of a Benthic Sargassum Population to Increased Temperatures: Decline in Non-Photochemical Quenching of Chlorophyll a Fluorescence (NPQ) Precedes That of Maximum Quantum Yield of PSII
Source: Plants (Basel). 2025 Mar 1;14(5):759. doi: 10.3390/plants14050759 (PMC11901439; doi:10.3390/plants14050759)
Supplement: Supplementary file 1 [file plants-14-00759-s001.zip › PLANTS Supplementary Material, Table S2.pdf]

Response of a Benthic *Sargassum* Population to Increased Temperatures: Decline of Non-Photochemical Quenching of Chlorophyll a Fluorescence (NPQ) Precedes That of Maximum Quantum Yield of PSII

SUPPLEMENTARY MATERIAL

Table S2

Effect of temperature on the maximum quantum yield of *Sargassum natans* and *Padina gymnospora* following 20, 40, and 120 hours of culturing in the laboratory for five days at 25, 31, and 34ºC under 90 µmol photons.$m^{-2}.s^{-1}$ irradiance, presented in **Figure 4**.
Tukey Test: significant differences between hours in bold, p<0.05. df= degrees of freedom.

Two-way Anova for repeated measures: Factor 1= temperature; Factor 2= exposure time

- *Padina gymnospora*

	Degrees of freedom	F values	Pr
temperature	2	0.316	0.732
time	3	0.727	0.547
Temperature x time	6	1.052	0.421
Residuals	21		

- *Sargassum natans*

	Degrees of freedom	F values	Pr
temperature	2	1.618	0.222
time	3	107.387	**6.70^{e-13}**
Temperature x time	6	54.313	**1.04^{e-11}**

Contrasts	p-values
Temperature = 25 ^{0}C	
0 x 20 min	**0.0074**
0 x 40 min	**0.0095**
0 x 120 min	0.1177
20 x 40 min	0.9995
20 x 120 min	0.5714
40 x 120 min	0.6377
Temperature = 31 ^{0}C	
0 x 20 min	1.0000
0 x 40 min	0.5254
0 x 120 min	**0.0050**
20 x 40 min	0.5189
20 x 120 min	**0.0048**
40 x 120 min	0.0977
Temperature = 34 ^{0}C	
0 x 20 min	**<0.0001**
0 x 40 min	**<0.0001**
0 x 120 min	**<0.0001**
20 x 40 min	**0.0001**
20 x 120 min	**<0.0001**
40 x 120 min	**<0.0001**
