# Supplementary material for: Response of a Benthic Sargassum Population to Increased Temperatures: Decline in Non-Photochemical Quenching of Chlorophyll a Fluorescence (NPQ) Precedes That of Maximum Quantum Yield of PSII
Source: Plants (Basel). 2025 Mar 1;14(5):759. doi: 10.3390/plants14050759 (PMC11901439; doi:10.3390/plants14050759)
Supplement: Supplementary file 1 [file plants-14-00759-s001.zip › PLANTS Supplementary Material, Table S3.pdf]

Response of a Benthic *Sargassum* Population to Increased Temperatures: Decline of Non-Photochemical Quenching of Chlorophyll a Fluorescence (NPQ) Precedes That of Maximum Quantum Yield of PSII

SUPPLEMENTARY MATERIAL

Table S3

Maximum quantum yield (F_v/F_m) and complementary quantum yields (Y_{II}, Y_{NPQ} and Y_{NO}) of *Sargassum natans* and *Padina gymnospora* obtained in the laboratory assay (presented in Figures 4, 6 and 7). For F_v/F_m, % indicates the proportion of the value measured at each temperature (25, 31, 34ºC) and exposure times (20, 40, 120 h) in relation to the initial measurement (0 h). For Y_{II}, Y_{NPQ} and Y_{NO}, % indicates the proportion of the absorbed energy by the PSII antenna under 750 µmol photons.m^{-2}.s^{-1} PAR utilized by each complementary quantum yield. Plants were cultivated under 12 h photoperiod and 90 µmol photons.m^{-2}.s^{-1} irradiance. Means ±SD (n=3).

Temperature									
Exposure time (h)	**25°C**			**31°C**			**34°C**		
	20	40	120	20	40	120	20	40	120
Sargassum natans									
F_v/F_m	0.627 ± 0.042	0.630 ± 0.016	0.666 ± 0.015	0.741 ± 0.015	0.698 ± 0.051	0.624 ±0.086	0.461 ± 0.028	0.274 ± 0.012	*
	85.0%	85.0%	90.2%	100.0%	94.0%	84.0%	63.0%	37.4%	
Y_{II}	0.098 ± 0.012	0.113 ± 0.003	0.119 ± 0.003	0.111 ± 0.008	0.119 ± 0.030	0.091 ±0.006	0.044 ± 0.013	0.006 ± 0.005	*
	9.8%	11.3%	11.9%	11.1%	11.9%	9.1%	4.4%	0.6%	
Y_{NPQ}	0.464 ± 0.035	0.493 ± 0.04	0.445 ± 0.017	0.483 ± 0.036	0.454 ± 0.078	0.387 ± 0.014	0.092 ± 0.059	0.041 ± 0.030	*
	46.4%	49.3%	44.5%	48.3%	45.4%	38.7%	9.2%	4.1%	
Y_{NO}	0.438 ± 0.045	0.393 ±0.006	0.436 ± 0.017	0.405 ± 0.035	0.427 ± 0.061	0.522 ± 0.014	0.864 ± 0.054	0.953 ± 0.024	*
	43.8%	39.3%	43.6%	40.5%	42.7%	52.2%	86.4%	95.3%	
Padina gymnospora									
F_v/F_m	0.735 ± 0.035	0.716 ±0.007	0.688 ± 0.031	0.732 ± 0.042	0.718 ± 0.014	0.758 ± 0.004	0.733 ± 0.025	0.721 ± 0.031	0.720 ± 0.031
	100.7%	98.1%	94.2%	99.7%	97.8%	103.3%	100.1%	98.5%	98.4%
Y_{II}	0.081 ± 0.016	0.113 ± 0.098	0.100 ±0.011	0.100 ±0.005	0.134 ±0.014	0.109 ±0.008	0.097 ±0.020	0.090 ± 0.007	0.069 ±0.015
	8.1%	11.3%	10.0%	10.0%	13.4%	10.9%	9.7%	9.0%	6.9%
Y_{NPQ}	0.418 ± 0.030	0.379 ±0.091	0.337 ±0.058	0.336 ±0.074	0.467 ±0.024	0.408 ± 0.064	0.443 ± 0.064	0.388 ±0.012	0.484 ±087
	41.8%	37.9%	33.7%	33.6%	46.7%	40.8%	44.3%	38.8%	48.4%
Y_{NO}	0.501 ± 0.032	0.508 ± 0.010	0.562 ± 0.060	0.563 ± 0.073	0.399 ±0.025	0.484 ± 0.056	0.460 ± 0.059	0.521 ± 0.074	0.447 ± 0.062
	50.1%	50.8%	56.2%	56.3%	39.9%	48.4%	46.0%	52.1%	44.7%
